# Neuronal Membrane Disruption Occurs Late Following Diffuse Brain Trauma in Rats and Involves a Subpopulation of NeuN Negative Cortical Neurons

**DOI:** 10.3389/fneur.2019.01238

**Published:** 2019-11-22

**Authors:** Martina L. Hernandez, Todd Chatlos, Karen M. Gorse, Audrey D. Lafrenaye

**Affiliations:** ^1^Anatomy and Neurobiology, Virginia Commonwealth University, Richmond, VA, United States; ^2^Department of Neurology, University of California, Davis, Davis, CA, United States

**Keywords:** diffuse traumatic brain injury, membrane disruption, NeuN neuronal nuclei, mechanoporation, rat-brain

## Abstract

The repercussions of traumatic brain injury (TBI) endure years following the initial insult and involve chronic impairments/disabilities. Studies indicate that these morbidities stem from diffuse pathologies, however, knowledge regarding TBI-mediated diffuse pathologies, and in particular, diffuse neuronal membrane disruption, is limited. Membrane disruption has been shown to occur acutely following injury, primarily within neurons, however, the progression of TBI-induced membrane disruption remains undefined. Therefore, the current study investigated this pathology over a longer temporal profile from 6 h to 4 w following diffuse TBI induced using the central fluid percussion injury (CFPI) model in rats. To visualize membrane disruption, animals received an intracerebroventricular infusion of tagged cell-impermeable dextran 2 h prior to experimental endpoints at 6 h, 1 d, 3 d, 1 w, 2 w, or 4 w post-CFPI. The percentage of total neurons demonstrating dextran uptake, indicative of membrane disruption, was quantified within the lateral neocortex layers V and VI from 6 h to 4 w post-injury. We found that membrane disruption displayed a biphasic pattern, where nearly half of the neurons were membrane disrupted sub-acutely, from 6 h to 3 d post-TBI. At 1 w the membrane disrupted population was dramatically reduced to levels indistinguishable from sham controls. However, by 2 and 4 w following CFPI, approximately half of the neurons analyzed displayed membrane disruption. Moreover, our data revealed that a subset of these late membrane disrupted neurons were NeuN negative (NeuN-). Correlative western blot analyses, however, revealed no difference in NeuN protein expression in the lateral neocortex at any time following injury. Furthermore, the NeuN- membrane disrupted neurons did not co-label with traditional markers of astrocytes, microglia, oligodendrocytes, or NG2 cells. Immunohistochemistry against NeuN, paired with a hematoxylin and eosin counter-stain, was performed to quantify the possibility of overall NeuN+ neuronal loss following CFPI. A NeuN- population was observed consistently in both sham and injured animals regardless of time post-injury. These data suggest that there is a consistent subpopulation of NeuN- neurons within the lateral neocortex regardless of injury and that these NeuN- neurons are potentially more vulnerable to late membrane disruption. Better understanding of membrane disruption could provide insight into the mechanisms of diffuse pathology and lead to the discovery of novel treatments for TBI.

## Introduction

Traumatic brain injury (TBI) is a continuing healthcare problem, resulting in mortality and long-lasting morbidities that coalesce into various impairments and/or disabilities (1). In the United States, ~2.8 million people report suffering a TBI annually and this number is likely an underestimate since TBI often goes unreported (2, 3). Various studies have demonstrated links between disabilities/impairments and diffuse pathologies (4). However, diffuse pathologies are highly heterogeneous (5–7). Additionally, while substantial progress has been made in understanding the pathophysiology of TBI-induced focal injuries, knowledge about diffuse pathologies following TBI remain limited.

One such pathology is diffuse membrane disruption, which is characterized by leaky somatic plasmalemma (5, 8, 9). Membrane disruption has been well-established to occur in various models of TBI, both *in vitro* using cell stretch and *in vivo* following focal brain and spinal cord injury as well as after diffuse TBI (5, 6, 10–18). These studies found that membrane disruption occurred upon physical impact (mechanoporation), as well as sub-acute membrane disruption, in which mechanical transduction is less likely to be directly instigating damage (5, 6, 10–14, 18, 19). Membrane disruption has primarily been evaluated in models of focal TBI, wherein the pathological progression is linked to cell death in the pericontusional lesion, however, less is known regarding the pathological progression of membrane disruption in a diffuse model of TBI, in which cell death is not observed (5, 10, 12, 18, 20, 21). We previously demonstrated that neuronal membrane disruption is induced in layers V and VI of the lateral neocortex hours following diffuse central fluid percussion injury (CFPI). This diffuse membrane disruption did not progress to cell death, but could be exacerbated by secondary insults, such as increased intracranial pressure, which did precipitate neuronal loss (10, 18). However, the natural progression of this pathology to later time points following experimental diffuse TBI has not been explored previously.

Neuronal Nuclei (NeuN) is an RNA-binding protein potentially involved in neuronal maturation and is exclusively expressed by post-mitotic neurons throughout the brain and spinal cord (22–24). Due to this neuron-specific expression, NeuN has been widely adopted as a ubiquitous marker for mature neurons throughout the central nervous system (25, 26). However, there are subsets of neurons that never express NeuN, including cerebellar Purkinje cells, olfactory mitral cells, retinal photoreceptors, subsets of interneurons, and inner granule cells (23, 24). Studies have also indicated that damaged neurons could reduce NeuN expression upon reversion to a less mature growth-permissive state (27, 28). The expression of NeuN within the diffusely membrane disrupted population of cortical neurons, however, has not previously been assessed.

It has been theorized that membrane disruption would inevitably progress to cell death as mechanoporation has been demonstrated to precipitate uncontrolled calcium influx, ATP dysregulation, and eventual cell death *in vitro* (29, 30). However, neurons sustaining membrane disruption minutes to hours post-diffuse TBI have also been demonstrated to be capable of membrane resealing and cell survival, making membrane disruption a targetable pathology for therapeutic intervention (5, 10–13, 18). Therefore, in this study we sought to establish a temporal profile for cortical membrane disruption following diffuse brain injury.

Throughout this study we found cortical neurons sustaining membrane disruption, weeks following TBI, that appear temporally distinct from the disrupted populations sustaining membrane disruption hours to days post-CFPI. Furthermore, we also discovered a subpopulation of NeuN negative (NeuN-) membrane disrupted neurons that were most apparent 2 w post-injury. Interestingly, we found that there was a consistently present NeuN- subpopulation diffusely distributed throughout layers V and VI of the lateral neocortex regardless of injury. Together, the findings presented below highlight the complexity of diffuse neuronal membrane disruption. Better understanding of membrane disruption could provide insight into the mechanisms of diffuse pathology and lead to the discovery of novel treatments following TBI.

## Methods

### Animals

Experiments were conducted using protocols in accordance with the Virginia Commonwealth University institutional ethical guidelines concerning the care and use of laboratory animals (Institutional Animal Care and Use Committee, Virginia Commonwealth University), which adhere to regulations including, but not limited to, those set forth in the Guide for the Care and Use of Laboratory Animals, 8th Edition (National Research Council). Animals were housed in individual cages on a 12 h light-dark cycle with free access to food and water. Adult male Sprague-Dawley rats, *n* = 66 weighing 350–450 g were used for this study. Any animal that lost more than 20% of their pre-injury body weight or precipitated gross brain pathology (contusion, subdural hematoma, or gross tissue loss) was excluded from analysis. No animals met exclusion criteria in this study. Animal injury state and survival time point were randomly determined using a random number generator on the day of surgery. All surgeries were conducted by the same surgeon during the same times of day to reduce variability.

### Surgical Preparation and Injury Induction

Animals were anesthetized with 4% isoflurane in 30% O_2_ and 70% N_2_O then intubated and ventilated with 2% isoflurane in 30% O_2_ and 70% N_2_O throughout the duration of the surgery, injury, and post-injury physiological monitoring. Heart rate, respiratory rate, and blood oxygenation were monitored via a hind-paw pulse oximetry sensor (STARR Life Sciences, Oakmont, PA) for the duration of anesthesia, except during the induction of injury. Body temperature was maintained at 37°C with a rectal thermometer connected to a feedback-controlled heating pad (Harvard Apparatus, Holliston, MA). All animals were placed in a stereotaxic frame (David Kopf Instruments, Tujunga, CA). A midline incision was made, and a 4.8 mm circular craniectomy was made along the sagittal suture midway between bregma and lambda for injury induction. A 2 mm burr hole was also drilled in the left parietal bone overlying the left lateral ventricle (0.8 mm posterior, 1.3 mm lateral, and 2.5- 3 mm ventral to bregma) through which a 25-gauge needle, connected to a pressure transducer and micro infusion pump (11 Elite syringe pump; Harvard Apparatus) via sterile saline filled PE50 tubing, was placed into the left lateral ventricle. Appropriate placement was verified via a 1.3 μl/min infusion of sterile saline within the closed fluid-pressure system during needle placement (31). The needle was held in the ventricle for at least 5 min to record pre-injury intracranial pressure (ICP). After the 5-min reading, the needle was slowly removed and the burr hole was covered with bone wax before preparation for sham or CFPI (10, 32). Briefly, a Leur-Loc syringe hub was affixed to the craniectomy site and dental acrylic (methyl-methacrylate; Hygenic Corp., Akron, OH) was applied around the hub and allowed to harden. Anesthetized animals were removed from the stereotaxic frame and injured at a magnitude of 2.05 ± 0.15 atmospheres (*F*_5, 24_ = 0.565, *p* = 0.726; 6 h = 2.03 ± 0.08; 1 d = 2.06 ± 0.1; 3 d = 2.07 ± 0.05; 1 w = 2.02 ± 0.8; 2 w = 2.1 ± 0.04; 4 w = 2.05 ± 0.1) and duration of ~22 msec. The pressure pulse was measured by a transducer affixed to the injury device and displayed on an oscilloscope (Tektronix, Beaverton, OR). Immediately after the injury, the animal was reconnected to the ventilator and physiologic monitoring device and the hub and dental acrylic were removed en bloc. Gelfoam was placed over the craniectomy/injury site and the scalp was sutured. The animal was then replaced in the stereotaxic device and the ICP probe was reinserted into the lateral ventricle, as described above, for post-injury ICP monitoring. The animals were then allowed to recover and were returned to clean home cages. Identical surgical procedures were followed for sham-injured animals, without release of the pendulum to induce injury.

### Tracer Infusion

Two hours prior to sacrifice, tagged dextran (40 mg/ml in sterile 0.9% saline; ~1.6 mg/kg) was infused into the lateral ventricle as described previously (18). Briefly, 15 μl of 10 kDa dextran conjugated to either 488-Alexa Fluor (Cat#: D22910, Invitrogen, Carlsbad, CA), 568-Alexa-Fluor (Cat#: D22912; Invitrogen, Carlsbad, CA), or biotin (Cat#: D1956; Invitrogen, Carlsbad, CA) was infused into the left lateral ventricle at 0.5–1.3 μl/min, with continuous ICP monitoring. To avoid bias caused by differences in fluorescent signal detectability animals were randomly assigned a tag (Alexa 488, Alexa 568 or biotin). The tracer was allowed to diffuse throughout the parenchyma for 2 h prior to transcardial perfusion at 6 h, 1 d, 3 d, 1 w, 2 w, or 4 w post-sham or CFPI.

### Tissue Processing

At appropriate time-points between 6 h and 4 w post-injury, the animals were injected with 150 mg/kg euthanasia-III solution (Henry Schein, Dublin, OH), then underwent transcardial perfusion with cold 0.9% saline. Lateral neocortices were dissected from the right hemisphere of the brain for molecular assessments of protein expression followed by a switch in transcardial perfusate to 4% paraformaldehyde/0.2% gluteraldehyde in Millonig's buffer (136 mM sodium phosphate monobasic/109 mM sodium hydroxide) to fix the left side of the brain for subsequent immunohistochemical or electron microscopic (EM) processing and analysis. After transcardial perfusion, the brains were removed, post-fixed for 24–48 h, then sectioned coronally in 0.1 M phosphate buffer with a vibratome (Leica, Banockburn, IL) at a thickness of 40 μm from bregma to 4.0 mm posterior to bregma. Sections were collected serially in 12 well-plates and stored in Millonig's buffer at 4°C. A random starting well (wells 1–12) was selected using a random number generator and four serial sections, each 480 μm apart, were used for histological analyses. All histological analyses were restricted to layers V and VI of the lateral somatosensory neocortex extending from the area lateral to CA1 to the area lateral to CA3 of the hippocampus.

### Western Blotting

Lateral neocortices of sham *n* = 6 (*n* = 1/time point) and TBI rats *n* = 4/time point were homogenized in NP40 Buffer (150 mM NaCl, 50 mM Tris pH 8.0, 1% Triton) and protease inhibitor cocktail (AEBSF 10.4 mM, Aprotinin 8 μM, Bestatin 400 μM, E-64 140 μM, Leupeptin 8 μM, Pepstatin A 150 μM, Cat#: P8340, Sigma, Saint Louis, MO). Protein concentrations were measured using a NanoDrop Lite (Thermo Fisher Scientific, Wilmington, DE). Protein (20 μg) was boiled for 10 min in 50 mM dithiothreitol (Cat#: 1610610; Bio-Rad; Hercules, CA), 2x Laemelli loading buffer (Cat#: 1610737; Bio-Rad; Hercules, CA) and run at 200 Volts for 30 min on Mini-PROTEAN TGX Stain-Free 4–20% precast polyacrylamide gels (Cat#: 4568096; Bio-Rad, Hercules, CA). Protein was transferred onto 0.45 μm PVDF membranes using Bio-Rad Transblot Turbo transfer system using mixed molecular weight manufacturer setting (1.3–2.5 Amps, 25 Volts for 7 min). Western blotting was done on an iBind flex apparatus (Invitrogen, Carlsbad, CA) using primary antibody rabbit anti-NeuN (1:2000; Cat#: ab104225; Abcam; Cambridge, MA) and anti-rabbit-HRP secondary antibody (1:5000; Cat#: 111-030-003; Jackson Laboratories, West Grove, PA) (33). Total protein (Stain Free) and chemiluminescent images were taken on a ChemiDoc imaging system (BioRad). Densitometric analysis was done in ImageJ (National Institutes of Health; Bethesda, MD) and all NeuN protein bands were normalized to total protein and sham controls.

### Membrane Disruption Analysis

Consistent with previous studies, we assessed the potential for neuronal membrane disruption via the utilization of 10 kDa dextrans, which are impermeable to cells with intact membranes (5, 6, 13, 18, 29, 34). Fluorescently tagged dextran-containing cells, indicative of membrane perturbation, could be visualized via confocal microscopy without further processing, however, biotin-conjugated dextran required immunolabeling for visualization. Tissue sections from sham *n* = 7 and TBI animals *n* = 5 per time point were blocked with 5% normal goat serum (NGS) or 5% normal horse serum (NHS), 2% bovine serum albumin (BSA), and permeabilized with 1.5% triton-X for 2 h. This was followed by immunolabeling using primary antibodies mouse anti-NeuN (1:500–700; Cat#: MAB377; MilliporeSigma; Temecula, CA) and Goat anti-biotin (1:2000; Cat#: 31852; Thermo Scientific, Rockford, IL). Secondary antibodies Alexa-568 conjugated goat anti-mouse (1:700; Cat#: A11004; Life Technologies, Carlsbad, CA) and Alexa-488 conjugated donkey anti-goat (1:700; Cat#: A11055; Life Technologies, Carlsbad, CA) and the tissue was mounted onto slides using Vectashield hardset mounting medium with 4′,6-diamidino-2-phenylindole (DAPI) (Cat#: H-1500; Vector Laboratories, Burlingame, CA). Sections were analyzed by confocal microscopy using a Zeiss LSM 710 System (Carl Zeiss). Quantitative analysis was performed as described previously (10). Briefly, confocal images of the left neocortical region of interest were taken at 40X magnification in a systematically random fashion by a blinded investigator using DAPI labeling to verify focus. Image acquisition settings were held constant for comparable regions (layer V or VI) for all groups analyzed. Analyses of neurons exhibiting dextran uptake were performed using the ImageJ colocalization finder plugin (overlap coefficient ≥0.9) and traditional cell counting. Dextran containing neurons were quantified for each image and averaged for each animal.

### Glial Analysis

Tissue slices from 2 w post-TBI animals *n* = 5, including sham *n* = 1, containing tagged dextrans were blocked with 5% NGS, 2% BSA, and 1.5% triton-X for 2 h, then incubated with mouse anti glial fibrillary acidic protein (GFAP) (1:1000; Cat#: MAB3402; MilliporeSigma, Temecula, CA) in 5% NGS/2% BSA/ 0.5% triton-X overnight at 4°C. The following day, the tissue was rinsed in 1% NGS/1%BSA/0.2% triton-X, then incubated in goat anti-mouse 568 (1:700; Cat. #: A11004; Life Technologies, Carlsbad, CA). Additional tissue slices were labeled for microglia using ionized calcium binding adaptor molecule 1 (Iba-1) and for oligodendrocytes via Adenomatous Polyposis Coli (APC/CC-1). Triple-labeled samples were prepared by blocking tissue in 5% NGS/2% BSA/1.5% triton-X for 2 h, then incubating with mouse anti-APC/CC-1 (1:200; Cat#: OP80 MilliporeSigma, Temecula, CA) and rabbit anti-Iba-1 (1:1000; Cat#: 19-19741; Wako Chemicals, Richmond, VA) overnight at 4°C. Tissue was then incubated with goat anti-mouse Alexa Fluor 568 and Alexa Fluor 633 conjugated goat anti-rabbit (1:700; Cat#: A21071; Invitrogen, Carlsbad, CA) for 2 h. Tissue probed for NG2 cells was blocked in 5% NGS/2% BSA/1.5% triton-X for 2 h, then incubated with rabbit anti-NG2 Chondroitin Sulfate Proteoglycan (1:200, Cat#: AB5320; MilliporeSigma, Temecula, CA) in 5% NGS/2% BSA/ 0.5% triton-X overnight at 4°C. The next day the tissue was rinsed then incubated with Alexa Fluor 633 conjugated goat anti-rabbit secondary antibody for 2 h. All labeled tissue was mounted using Vectashield hardset mounting medium with DAPI (Cat#: H-1500; Vector Laboratories, Burlingame, CA). Tissue with 488-conjugated dextrans, labeled for GFAP or CC-1 and Iba-1 or NG2 was visualized on a Zeiss LSM 710 system (Carl Zeiss, Oberkochen, Germany). To determine if dextran-containing cells also labeled for glia, co-labeling with either GFAP, Iba-1, CC-1, or NG2 was assessed using a Zeiss 710 confocal microscope by an investigator blinded to animal group during image acquisition and through the analysis. Three micrographs/regions of interest (ROIs) for each section were taken using a 40x objective; two random sections were assessed for each animal. The total number of dextran-containing cells co-labeling with either GFAP, Iba-1, CC-1, or NG2 were counted by eye for each image in Zen (Carl Zeiss, Oberkochen, Germany). The number of dextran-containing glia per 0.44 mm^2^ was averaged for each animal and compared to sham.

### TUNEL Analysis

One section of tissue from each animal sham *n* = 6, 6 h *n* = 5, 1 d *n* = 6, 3 d *n* = 6, 1 w *n* = 5, 2 w *n* = 6, 4 w *n* = 5 were mounted on slides for TUNEL. All samples were incubated in proteinase K (1:25). The positive control was subjected to DNase I (2 U/μL; Cat#: M0303S; New England BioLabs; Ipswich, MA) prior to TUNEL. Slides were incubated in terminal deoxynucleotidyl transferase (TdT) reaction according to the Click-iT Plus TUNEL assay (Cat#: C10619; Invitrogen; Carlsbad, CA), except for the negative control, which received water instead. The Click-iT Plus TUNEL reaction cocktail containing Alexa Fluor 647 picolyl azide was used to label fragmented DNA as an indicator of cell death. All slides were blinded against animal group and images were taken on a Zeiss 710 confocal microscope holding the imaging settings consistent. For all images, the number of TUNEL+ cells were quantified by eye per 0.43 mm^2^.

### Preparation of Tissue for Light Microscopy for Neuronal Quantification

In preparation for light microscopy, tissue was labeled with mouse anti-NeuN (1:700; Cat#: MAB377; MilliporeSigma; Temecula, CA). Tissue slices were blocked with 5% NGS, followed by incubation with biotinylated goat anti-mouse (1:1000; Cat#: BA-9200; Vector Laboratories) secondary antibody. Sections were then incubated in avidin biotinylated enzyme complex using the Vectastain ABC kit (Vector Laboratories) followed by visualization with 0.05% diaminobenzidine/0.01% hydrogen peroxide/0.3% imidazole (DAB) in 0.1 M phosphate buffer. For light microscopy, tissue was mounted on gelatin-coated slides before dehydration and rehydration. Rehydrated tissue was incubated in Gills hematoxylin (Leica Biosystems) followed by bluing agent (Leica Biosystems) and three dips in 0.25% eosin Y/0.005% acetic acid/95% ethanol before sections were cleared through increasing concentrations of ethanol and cover-slipped with Permount (Thermo Fisher Scientific, Waltham, MA). Light micrographs were acquired with a Nikon Eclipse 800 microscope (Nikon, Tokyo, Japan) equipped with an Olympus DP71 camera (Olympus, Center Valley, PA). To evaluate numbers of total neuronal population following injury, four sections per animal (sham *n* = 6; 6 h *n* = 7; 1 d *n* = 4; 3 d *n* = 5; 1 w *n* = 5; 2 w *n* = 5; 4 w *n* = 4 animals) were stained with NeuN and H&E as described above. An investigator, blinded to animal group, imaged and analyzed all slides. Approximately 8–10 images spanning the lateral neocortex were taken per section in a systematically random fashion. Neurons were denoted by hematoxylin and eosin stained cell bodies that were at least 2x larger than the surrounding glial cells (10). All cell numbers were counted by eye and any cells that were morphologically determined to be neurons but didn't contain labeling for NeuN were denoted as NeuN-.

### Ultrastructural Assessment of Membrane Disrupted Neurons

In preparation for electron microscopic (EM) analysis, tissue was labeled with rabbit antibodies targeted to Alexa Fluor 488 (1:5000; Cat#: A11094; Invitrogen; Carlsbad, CA). Tissue slices were then blocked with 5% NGS, followed by incubation with biotinylated goat anti-rabbit (1:1000; Cat#: BA-5000; Vector Laboratories) secondary antibody. Sections were then incubated in avidin biotinylated enzyme complex using the Vectastain ABC kit (Vector Laboratories) followed by visualization with DAB in 0.1 M phosphate buffer. Tissue sections were osmicated, dehydrated, and embedded in epoxy resin on plastic slides. After resin curing, areas of interest were identified using light microscopy. These areas were removed, mounted on plastic studs, and 70 nm sections were cut and mounted on Formvar-coated slotted grids. The grids were stained in 5% uranyl acetate in 50% methanol and 0.5% lead citrate. Electron micrographs were imaged using a JEOL JEM 1230 transmission electron microscope equipped with an Orius SC1000 CCD cameras (Gatan, Pleasanton, CA).

### Statistical Analysis

Data were tested for normality prior to utilizing parametric or non-parametric assessments, which were conducted in SPSS (IBM Corporation, Armonk, NY). Animal numbers for each group were determined by an a priori power analysis using effect size and variability previously observed in the lab when assessing pathology between sham and injured groups using the CFPI model, an alpha = 0.05, and a power of 80%. One-way analysis of variance (ANOVA) and Bonferroni *post hoc* test were performed for all between group histological analyses. Non-normal data underwent non-parametric analysis Kruskal–Wallis mean rank sum testing to compare groups. Statistical significance was set to *p* < 0.05. Data are presented as mean ± standard error of the mean.

## Results

### Neuronal Membrane Disruption Is Biphasic and Extends Out to 4 Weeks Following CFPI

**Figure 1:**
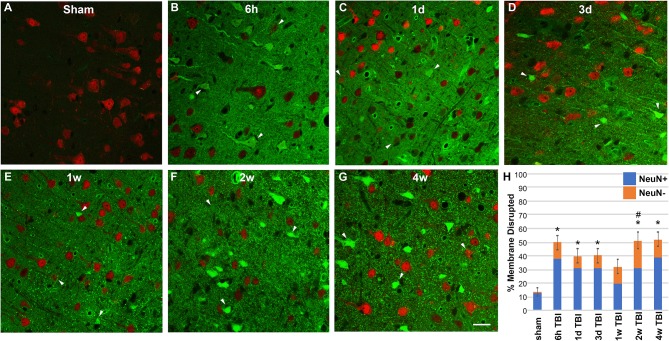
TBI-induced membrane disruption occurs biphasically and involves a NeuN negative subpopulation. **(A–G)** Representative photomicrographs of lateral neocortex from animals sustaining either **(A)** sham injury or **(B–G)** CFPI at **(B)** 6 h, **(C)** 1 d, **(D)** 3 d, **(E)** 1 w, **(F)** 2 w, and **(G)** 4 w post-injury. Neurons expressing NeuN are in red. Cells containing the cell-impermeable fluorescent-conjugated dextran (green) were considered membrane disrupted (arrow heads). **(H)** Bar graph depicting the mean percentage of membrane disrupted neurons per total number of neurons analyzed. Note that while hardly any membrane disruption was seen in shams, robust membrane disruption occurred both 6 h−3 d and 2–4 w post-CFPI. While the majority of membrane disrupted neurons expressed NeuN (NeuN+; blue bars), a population of membrane disrupted cells that were not labeled with NeuN (NeuN-, orange bars) emerged following CFPI. This NeuN- membrane disrupted subpopulation was significantly pronounced at 2 w post-CFPI. Data presented as mean ± S.E.M. *p < 0.05 percent of total membrane disrupted cells compared to sham, ^#^*p* < 0.05 percent NeuN- membrane disrupted cells compared to sham. Scale bar = 20 μm.

Based on our previous findings of substantial neuronal membrane disruption hours following CFPI without subsequent cell death, unless compounded by secondary insult, we evaluated the temporal progression of diffuse membrane disruption from hours to weeks post-injury [Figure 1; (10)]. As has been well-established, tagged 10 kDa dextrans that are normally excluded from cells with intact membranes can be reliably used to identify membrane disrupted neurons after injury both *in vitro* and *in vivo* (6, 9, 11, 13, 18, 19). Therefore, we infused tagged dextran intracerebroventricularly (ICV) prior to sacrifice at various time points ranging from 6 h to 4 w post-CFPI. Cells containing dextran were considered membrane disrupted and were quantified throughout layers V and VI of the lateral neocortex. Within sham animals (Figure 1A), membrane disruption was rarely detected (13.88% ± 3.25 total neurons), however, rats sustaining TBI demonstrated significant membrane disruption [Figure 1; one way-ANOVA *F*_6, 29_ = 8.20 *p* = 3.1 × 10^−5^]. At 6 h following injury (Figure 1B), over half of the total neurons assessed demonstrated membrane disruption (52.29% ± 3.73 total neurons; *p* = 2.34 × 10^−4^ vs. sham). At 1 and 3 d post-injury (Figures 1C,D), membrane disruption remained significantly elevated (1 d: 37.94% ± 5.66, *p* = 0.013 vs. sham and 3 d: 39.23% ± 6.39 total neurons, *p* = 0.012 vs. sham, respectively). At 1 w post-CFPI (Figure 1E), though, the number of neurons that were disrupted declined to levels indistinguishable from sham (32.32% ± 4.99, *p* = 0.233 vs. sham). However, late (Figures 1F,G) membrane disruption resurged to levels similar to that observed hours-days post-CFPI (2 w: 51.19% ± 6.08, *p* = 1.37 × 10^−4^ vs. sham and 4 w: 52.02% ± 5.39, *p* = 9.80 × 10^−5^ vs. sham). It was also noted that the intensity of tagged dextran within the parenchyma was drastically increased in injured animals as compared to sham (Figure 1).

### Chronic Membrane Disruption Is Associated With a Transient Shift Toward a NeuN Negative Phenotype

Membrane disrupted cells that didn't label with NeuN were also quantified in sham controls and at 6 h−4 w post-TBI (Figure 1). Very few NeuN- membrane disrupted cells were present in sham injured rats (1.10 ± 0.27% of total neurons; Figure 1A). However, a NeuN- membrane disrupted subpopulation was present within the lateral neocortex of animals following CFPI [one way-ANOVA *F*_6, 29_ = 0.604, *p* = 0.020]. While the percent of total membrane disrupted cells that displayed this NeuN- phenotype were not significantly different from sham sub-acutely (6 h = 12.94 ± 3.07%; 1 d = 7.70 ± 1.861%; 3 d = 7.56 ± 2.09%; 1 w = 12.53 ± 3.30%), or at the 4 w time point (13.11 ± 4.19%), at 2 w post-injury there was a significant shift in the late membrane disrupted population toward a NeuN- phenotype (19.99 ± 6.25%, *p* = 0.007 vs. sham).

### NeuN Negative Membrane Disrupted Cells Are Not Glia

**Figure 2:**
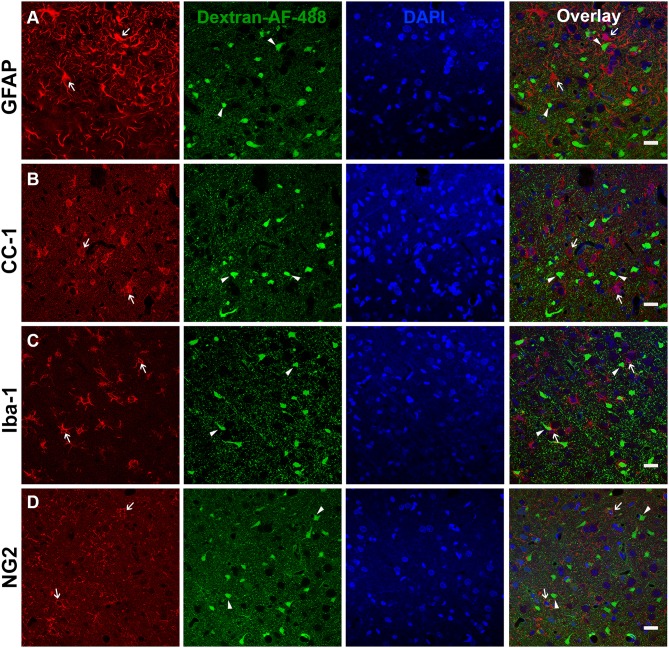
Glia do not appear to be membrane disrupted following CFPI. Representative photomicrographs of the lateral neocortex 2 w following CFPI labeled with **(A)** GFAP (astrocytes), **(B)** CC-1/APC (oligodendroglia), **(C)** Iba-1 (microglia), and **(D)** NG2 (NG2 cells) (far left panel; red). Membrane disrupted cells containing Alexa-488 conjugated dextran (green) are represented in the second panel. The third panel in blue are DAPI labeled nuclei. The last panel is the overlay images. Arrows depicted cells labeled with each representative marker in relation to the membrane disrupted cells (arrowheads). Note that there were no indications of glial cells sustaining membrane disruption at 2 w post-injury. Scale bar = 20 μm.

The presence of a transient shift toward this NeuN- subpopulation of late membrane disrupted cells indicated the possibility of passing glial membrane disruption. Therefore, 2 w post-injury tissue was probed with various glial markers including GFAP for astrocytes, CC-1 for oligodendrocytes, Iba-1 for microglia, and NG2 for NG2 cells, to assess possible overlap with the NeuN- late membrane disrupted subpopulation. There were no indications of substantial membrane disruption within astrocytes (0.37 membrane disrupted somas/0.44 mm^2^, Figure 2A), oligodendrocytes (0.10 membrane disrupted somas/0.44 mm^2^, Figure 2B), microglia (0.57 membrane disrupted somas/0.44 mm^2^, Figure 2C), or NG2 cells (1.23 membrane disrupted somas/0.44 mm^2^, Figure 2D), indicating that the NeuN- late membrane disrupted subpopulation are likely neurons.

### Cortical NeuN Protein Expression Does Not Change Following TBI

**Figure 3:**
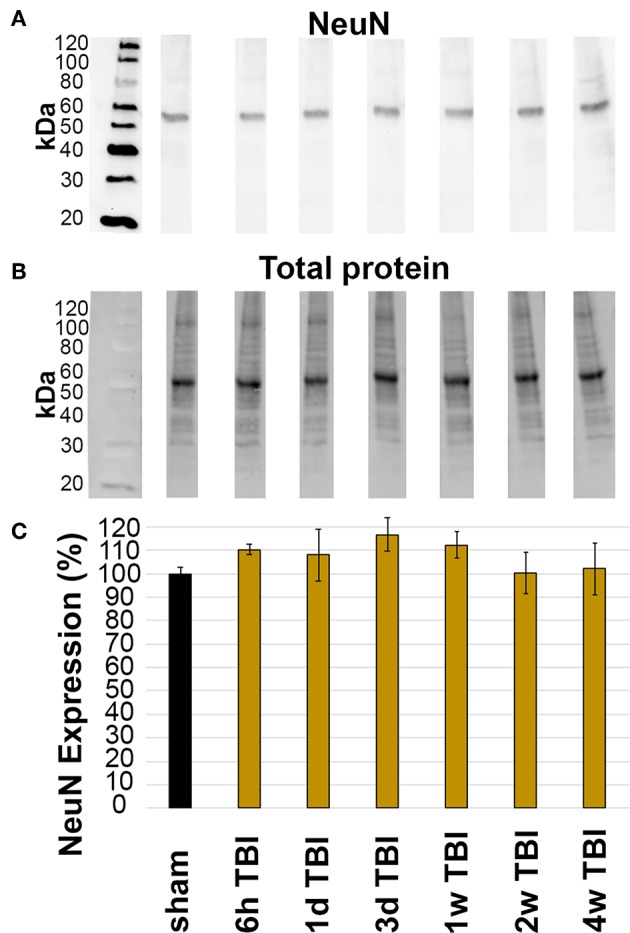
Expression of NeuN did not change in the lateral neocortex after TBI. Western blot analysis of **(A)** amount of NeuN protein (band at ~50 kDa) in lateral neocortical homogenates normalized to **(B)** total loaded protein. **(C)** Bar graph depicting the average percent change in NeuN expression in TBI rats at 6 h-4 w post-CFPI as compared to sham. Notice that there was no difference between sham and CFPI at any timepoint, denoted as mean ± S.E.M.

The overall expression of NeuN within the lateral neocortex was also evaluated for changes following CFPI. Investigation of NeuN protein expression was conducted in sham and TBI rats throughout the 6 h to 4 w post-injury time course. Western blot analysis revealed consistent NeuN protein levels in sham animals (100.00 ± 2.29%) and TBI animals at 6 h (110.28 ± 2.22% of sham), 1 d (108.11 ± 5.53% of sham), 3 d (116.58 ± 7.12% of sham), 1 w (112.22 ± 5.53% of sham), 2 w (100.26 ± 8.71% of sham), and 4 w (102.07 ± 11.09% of sham) post-CFPI (Figure 3; one-way ANOVA *F*_6, 23_ = 1.036, *p* = 0.428).

### Late Membrane Disruption Is Not Associated With Cell Death/Loss Post-CFPI

**Figure 4:**
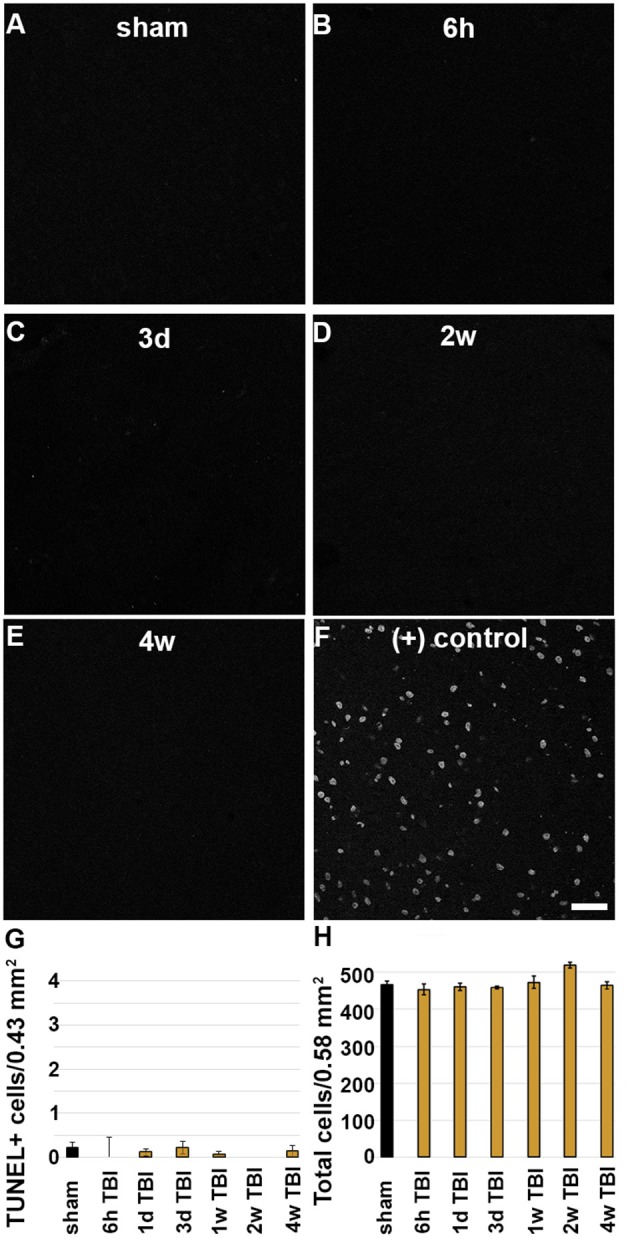
There is no indication of cell death in the lateral neocortex following CFPI. Representative micrographs depicting **(A)** sham and **(B)** 6 h, **(C)** 3 d, **(D)** 2 w, or **(E)** 4 w post-CFPI labeled for DNA damage using TUNEL. **(F)** TUNEL was verified using a DNase-treated positive control. Bar graphs depicting the average number of **(G)** TUNEL+ cells or **(H)** cells in general in layers V and VI of the lateral neocortex following sham (black bar) or CFPI (yellow bars) at 6 h−4 w post-injury. Note that there were very few TUNEL+ cells and no cell loss found in the lateral neocortex at any timepoint following injury. Scale bar = 50 μm.

As previous studies have shown that membrane disruption could lead to cell death, TUNEL was done to assess DNA damage indicative of late-stage cell death out to 4 w post-CFPI. In agreement with the literature and our previous study using the CFPI model in rats (18, 35–38) there was no difference in the number of TUNEL positive cells among sham animals compared to timepoints post-CFPI (Figures 4A–G; Kruskal Wallis mean rank comparison χ^2^ = 5.713, *p* = 0.456). Further assessment for potential cell loss was performed with H&E staining of sham and injured rats. Again, in agreement with the literature and our previous studies (10, 18, 35, 37, 38), there was no significant difference in overall neuronal numbers within layers V and VI of the lateral neocortex (Figure 4H; Kruskal Wallis mean rank comparison, χ^2^ = 11.580, *p* = 0.072).

**Figure 5:**
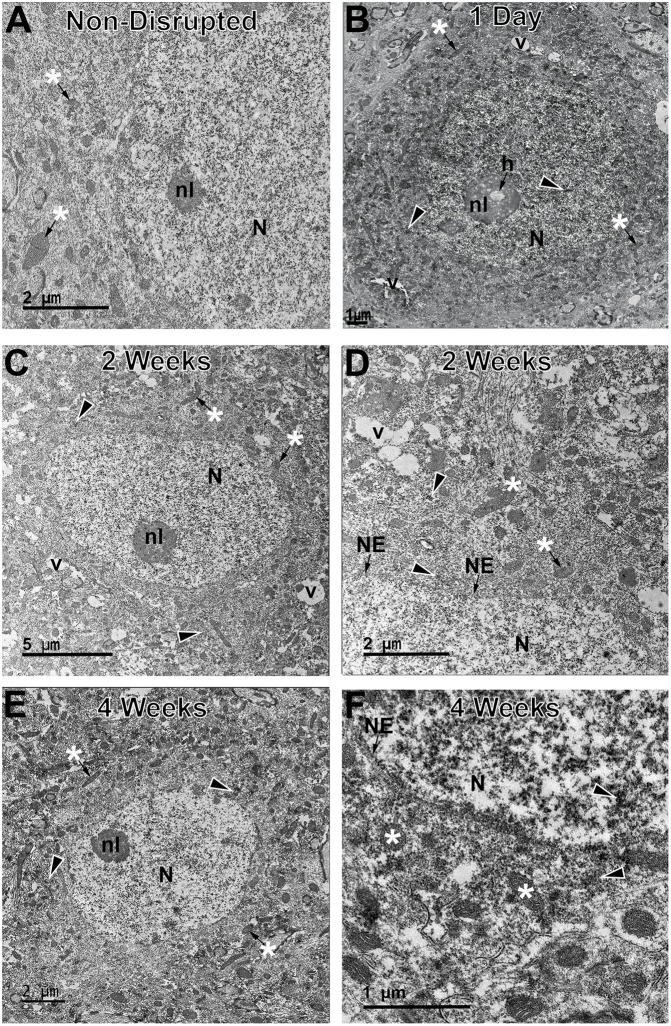
Neurons sustaining membrane disruption weeks post-injury display few signs of ultrastructural damage indicative of cell death. Representative electron micrographs of **(A)** non-membrane disrupted neurons and neurons sustaining membrane disruption sub-acutely at **(B)** 1 d and chronically at **(C,D)** 2 w and **(E,F)** 4 w post-injury. Some membrane disrupted neurons, particularly those that contained dextran (arrow heads) within both the cytoplasm and nucleus (N) at sub-acute time points, demonstrated ultrastructural changes, such as organelle vacuolization (v) and occasional holes (h) within the nucleolus (nl), indicative of enhanced nucleolar activity. However, the majority of late membrane disrupted neurons were largely unremarkable with no overt mitochondrial damage (*) and, in cases, intact nuclear envelope (NE). These features are better observed in the enlarged panels **(D,F)**.

Additionally, ultrastructural analysis of membrane disrupted neurons, as identified by immunoelectron microscopy against the dextran was used to further scrutinize potential subcellular alterations indicative of cell damage/death in both the sub-acutely and late membrane disrupted populations [(5, 18); Figure 5]. In agreement with our previous assessments, a subset of membrane disrupted neurons, particularly those with both plasmalemmal and nuclear membrane disruption at 6 h following CFPI, demonstrated some ultrastructural changes, such as organelle vacuolization, which indicated organelle pathology, and nucleolar holes, which potentially indicates altered nucleolar activity [(39); Figure 5B]. However, the majority of membrane disrupted neurons, did not demonstrate the ultrastructural characteristics of actively dying cells, including mitochondrial change, pyknosis, or extreme vacuolization, indicating that membrane disrupted neurons were not progressing to cell death (Figures 5C–F). The ultrastructural features of the membrane disrupted cells were also characteristic of neurons but not of glial cells (Figure 5).

### The Lateral Neocortex Contains a Subpopulation of NeuN Negative Neurons Regardless of Injury

**Figure 6:**
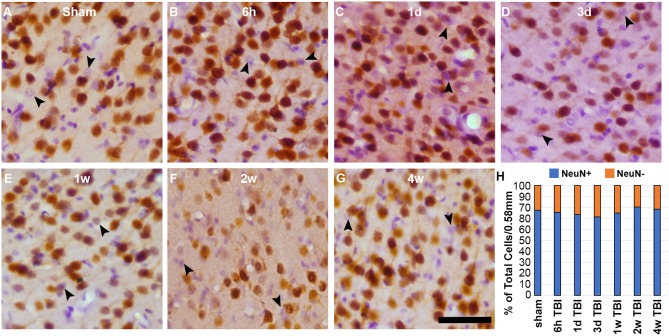
There is a consistent NeuN- subpopulation in layers V and VI of the lateral neocortex. Representative photomicrographs of cortices labeled with NeuN (brown) and counterstained with H&E (purple and pink respectively) from **(A)** sham-injured rats and rats at **(B)** 6 h, **(C)** 1 d, **(D)** 3 d, **(E)** 1 w, **(F)** 2 w, or **(G)** 4 w after sustaining a CFPI. Glial cells with smaller cell bodies and nuclei, were not counted. Neurons that do not label for NeuN (NeuN- neurons) are indicated by the arrow heads. **(H)** Stack graph depicting the percent of NeuN+ (blue bars) and NeuN- (orange bars) neurons in layers V and VI of the lateral neocortex in sham and CFPI rats. Interestingly, there was a consistent NeuN- subpopulation in all animals regardless of injury. Scale bar = 50 μm.

To assess the possibility that there was a general shift toward the NeuN- phenotype in the lateral neocortex at 2 w post-injury, regardless of membrane disruption, the total NeuN+ and NeuN- neuronal populations were quantified following CFPI using immunohistochemistry against NeuN paired with H&E staining (Figure 6). As expected, a sub-population of NeuN- neurons was apparent in layers V and VI of the lateral neocortex at 2 w post-injury (19.76 ± 2.75% total neurons/ROI). However, there was also a NeuN- subpopulation at 6 h (24.47 ± 1.91%), 1 d (26.51 ± 2.74%), 3 d (28.59 ± 2.25%), 1 w (25.06 ± 1.10%), and 4 w (21.59 ± 0.85%) following CFPI (Figure 6). Surprisingly, the proportion of neurons with a NeuN- phenotype was comparable with that seen within the lateral neocortex of sham controls [22.64 ± 1.83%; one-way ANOVA, *F*_6, 35_ = 1.930, *p* = 0.10], demonstrating that this NeuN- subpopulation of neurons is a consistent phenomenon in rats.

## Discussion

The current study demonstrates active neuronal membrane disruption weeks following diffuse TBI that appears to involve a shift in neuronal sub-population phenotype not seen in the membrane disrupted neurons at earlier time points. Specifically, membrane disruption following CFPI potentially occurs in a biphasic fashion, producing significant membrane disruption sub-acutely hours to days post-injury and weeks post-injury that are temporally segregated by a reduction at 1 w post-injury. We also observed a difference in the intensity of dextran within the parenchyma, potentially indicating additional issues with clearance mechanisms following CFPI. Based on *in vitro* studies demonstrating the lack of cellular dextran uptake in non-injured cultures despite the presence of dextran in the media, it is likely that while potential changes in dextran clearance and neuronal membrane disruption may be occurring concurrently, they do not appear to be interdependent (13, 40). The possible change in parenchymal clearance of dextran is an intriguing finding and will be further probed in future studies. Membrane disruption, however, has been demonstrated in various models of TBI, both *in vitro* using cell stretch models and *in vivo* following focal brain and spinal cord injury as well as after diffuse TBI (5, 6, 10–18).

Membrane disruption following trauma is primarily attributed to the mechanical force of injury directly altering membrane integrity, known as “mechanoporation” (5, 6, 11, 15, 16, 19, 40–42). Studies from the LaPlaca and Wanner labs found that mechanoporation, in both neurons and glia, is highly dynamic, occurring within seconds of injury and fluctuating for minutes post-insult (13, 15, 16, 42). Additional studies, both *in vitro* and in various animal models of injury, have demonstrated sub-acute neuronal membrane disruption occurring hours to days following the initial mechanical insult (5, 6, 10–13, 18, 19). This study, however, is the first to demonstrate a late phase of diffuse neuronal membrane disruption weeks following the initial mechanical injury. Based on the reduction in active membrane disruption at 1 w post-injury, it appears that late diffuse membrane disruption is not directly attendant to either mechanoporation or membrane disruption occurring hours to days following trauma.

It has been widely theorized that membrane disruption would inevitably progress to cell death. Uncontrolled calcium influx and dysregulation of ATP capable of acute cellular damage and progression to cell death has been demonstrated to occur immediately following mechanoporation *in vitro* (16, 19, 29, 40). Following focal injury in a mouse model of TBI all mechanoporated and sub-acutely membrane disrupted neurons labeled with the membrane impermeable irreversible DNA binder, propidium iodide, were lost within the first week of injury, supporting the idea that membrane disruption is a terminal cellular pathology (20). However, as focal and diffuse pathological progressions are distinct, and based on our previous and current findings, it appears that a different progression is occurring in the sub-acutely membrane disrupted populations following diffuse TBI as compared to the progression of these same cells in focal TBI models [Figure 1; (18, 43)]. In this study, the unchanged total neuronal count throughout the temporal profile post-injury (Figure 4) and lack of ultrastructural characteristics indicative of late-stage apoptosis or necrosis (Figure 5), demonstrate that neurons are not dying in the lateral neocortex up to 4 w following CFPI. The lack of cell death is in alignment with other studies utilizing the CFPI model of diffuse TBI (35–37). Although these membrane disrupted neurons do not appear to progress to cell death following diffuse TBI alone, our previous data suggests that these cells are potentially more susceptible to secondary insults, which, paired with diffuse TBI, can result in significant cell loss (10). A possible mechanism for this increased susceptibility of diffusely membrane disrupted neurons is Cathepsin B mislocalization, as indicated by our previous studies, however, further investigation into this possibility is needed (18).

The occurrence of membrane disruption both hours to days and weeks to a month post-CFPI with a significant reduction in active membrane disruption visualized at 1 w post-injury, indicates that diffuse neuronal membrane disruption occurs biphasically. As the survival time points were randomly assigned we cannot ignore the possibility that the reduction in membrane disruption at 1 w post-CFPI may be an artifact of slightly reduced injury intensity in animals survived to that end point. An alternative possibility is that at least sub-acutely membrane disrupted neurons undergo membrane resealing following diffuse TBI (Figure 1). Membrane resealing of mechanoporated and sub-acutely membrane disrupted neurons has been identified in various models using multiple membrane-impermeable tracers administered at pre and various post-injury time-points (5, 10–13, 18). Heterogeneity within the mechanoporated population, in which neighboring neurons demonstrated variable levels of susceptibility to membrane disruption and capacity for subsequent membrane resealing was also seen *in vitro* (42). Therefore, it is highly likely that the sub-acutely membrane disrupted neurons in the current study retain the capability to reseal their membranes, which may explain the reduction in membrane disruption seen at 1 w following CFPI.

Moreover, we also observed a significant increase in the proportion of late membrane disrupted neurons that lacked expression of the traditional mature neuronal marker, NeuN (Figure 1). Previous studies have demonstrated that glia, and specifically astrocytes, are susceptible to membrane disruption within neural-astrocyte co-cultures and enriched astrocyte cultures following stretch injury (13, 15, 16). However, *in vivo* this has only been recapitulated in a model of spinal cord injury in swine (15) but was not observed following focal brain injury in rodents (42). In agreement with the previous rodent brain injury study, we did not observe any significant glial membrane disruption at any time point following CFPI (Figure 2). This may be due to species differences, as we have previously reported species-dependent TBI-induced glial alterations (38). It could also be due to the ability of astrocytes to rapidly reseal their membranes, as demonstrated *in vitro* (42). However, ultimately our findings demonstrate that the NeuN- subpopulation of membrane disrupted cells are most likely neuronal.

In various studies NeuN was shown to be expressed exclusively by post-mitotic neurons throughout the brain and spinal cord (23, 24). Due to this neuron-specific expression, NeuN has been widely used as a marker of mature neurons within the central nervous system and has been utilized to assess the number of neurons in various brain regions during development and following injury/pathologies (23, 25, 26, 33, 44–47). There are, however, subsets of neurons that don't express NeuN, including cerebellar Purkinje cells, olfactory mitral cells, retinal photoreceptor, and inner granule cells as well as some neurons in the superchiasmatic nucleus and substantia nigra (23, 24, 46, 48). There is a possibility that the NeuN- neuronal subpopulation belongs to a certain NeuN- neuronal subtype; a possibility that will be thoroughly investigated in future studies. To our knowledge, however, this is the first study to quantify NeuN- cells morphologically identified as neurons within the adult rat cortex following diffuse brain injury. Surprisingly, we found a consistent neuronal subpopulation exhibiting a NeuN- phenotype that were diffusely dispersed throughout layers V and VI of the lateral neocortex. This NeuN- subpopulation comprised ~30% of the total lateral cortical neurons identified in both sham and injured animals and was consistent from hours to weeks post-CFPI (Figure 6). Taken together with the significant increase in the membrane disrupted NeuN- subpopulation 2 w post-CFPI, this could indicate that NeuN- neurons within the cortex are more susceptible to late, but not sub-acute membrane disruption. It is also possible that NeuN- neurons are particularly sensitive to injury intensity, as the 2 w survival group had a slightly higher level of injury. The reduction in the NeuN- subpopulation to levels consistent with sham and sub-acutely membrane disrupted neurons by 4 w post-injury further suggests that at least some of these susceptible NeuN- neurons undergo membrane resealing.

Reduction of NeuN expression without correlative cell death could also indicate neuronal compromise and/or issues with proper neuronal maturation, as was proposed following sudden infant death syndrome and/or sudden intrauterine unexplained death syndrome (49). NeuN, which was identified as RNA-binding Fox-3 (Rbfox3), has been theorized to play a role in neuronal development by regulating neuron-specific alternative gene splicing (50, 51). Following ischemic injury, Ünal-Çevik and colleagues observed reduced NeuN labeling without correlative changes in NeuN protein expression, however, they did not quantify the NeuN- population (52). Another study found a population of medullary respiratory neurons displaying a NeuN- phenotype following a cervical spinal cord hemisection that co-expressed with markers of axonal regeneration, suggesting that these NeuN- neurons could play an ameliorative role following injury (28). Therefore, it is possible that the NeuN- population could represent reversion to a growth-permissive immature state following diffuse TBI, however, that is currently speculative and would require additional follow-up studies to rigorously evaluate this possibility.

Taken together, the findings presented here highlight the complexity of TBI-induced neuronal membrane disruption. Neuronal membrane disruption can occur much later than previously thought and involves subpopulations of cortical neurons demonstrating both NeuN+ and NeuN- phenotypes. Due to its biphasic nature, late membrane disruption appears to be distinct from mechanoporation and sub-acute membrane disruption. Additionally, this study indicates that membrane disruption, either acute or late, maintains the capacity for membrane repair and therefore potential therapeutic amelioration over a broad post-injury timeframe. Gaining a better understanding of TBI-induced membrane disruption could lead to the discovery of novel treatments to reduce morbidity associated with diffuse pathology in the human population.

## Data Availability Statement

The datasets generated for this study are available on request to the corresponding author.

## Ethics Statement

The animal study was reviewed and approved by Institutional Animal Care and Use Committee, Virginia Commonwealth University.

## Author Contributions

MH processed and carried out the analysis of light microscopic and confocal microscopic analyses, western assessments, and wrote the manuscript. TC and KG carried out analysis of confocal images. AL carried out the confocal microscopic and ultrastructural analyses, conceived, designed and coordinated the study, and wrote the manuscript.

### Conflict of Interest

The authors declare that the research was conducted in the absence of any commercial or financial relationships that could be construed as a potential conflict of interest.
